# Determining the willingness of Australians to export their corneas on death

**DOI:** 10.1371/journal.pone.0246622

**Published:** 2021-02-19

**Authors:** Heather M. Machin, Lisa Buckland, Christine Critchley, Steven Wiffen, Gerard Sutton, Paul N. Baird

**Affiliations:** 1 Lions Eye Donation Service, Centre for Eye Research Australia, Royal Victorian Eye and Ear Hospital, East Melbourne, Australia; 2 University of Melbourne, Melbourne, Australia; 3 Lions Eye Bank of Western Australia, Lions Eye Institute, Nedlands, Australia; 4 Swinburne University of Technology, Melbourne, Australia; 5 Centre for Law and Genetics, University of Tasmania, Hobart, Australia; 6 The University of Sydney, Save Sight Institute, Discipline of Ophthalmology, Sydney Medical School, Sydney, New South Wales, Australia; 7 The University of Technology Sydney, Graduate School of Health, Ultimo, New South Wales, Australia; Save Sight Institute, AUSTRALIA

## Abstract

**Background:**

12.7 million people await a corneal transplant, but 53% are without access to corneal tissue. Sharing corneal tissue across nations can provide some access, however the willingness of export populations, like Australians, to export their donation on death, has never been evaluated. Our research samples the Australian population, determining their willingness to export.

**Materials and method:**

We conducted e-surveys. N = 1044 Australians participated. The sample represented the Australian population, based on population demographics. Chi-Square and bivariate correlation coefficients examined associations between categorical variables, with a sample size of N = 1044, power of 0.80, and alpha of p = 0.05. Outcome measures were based on population sampling, by exploring willingness export, through the e-survey method.

**Results:**

38% (n = 397) of respondents said yes to exportation, 23.8% (n = 248) said no, and 38.2% (n = 399) were undecided. We found no relationship between willingness to export and general demographics, though those registered on the Donatelife Register (p = < .001), and those already willing to donate their eyes (p = < .001) were significantly more willing to export.

**Discussion:**

More Australians are willing to export their corneas than not, though a significant portion remain undecided. The Donatelife Register, and donation awareness, are key components of respondent decision making. Therefore, the provision of information about exportation prior to, and at the point-of-donation, is essential for assisting Australian’s to decide to export or not. Further examination and development of consent-for-export systems are necessary before routine exportation is undertaken.

## Introduction

Nations without routine local access to corneal tissue rely on transnational activity (export/import) as a method of obtaining corneal tissue for corneal transplantation in their nation [1,2]. The activity can occur legally (e.g., government and/or sector planned or permitted) or illegally (e.g. black market or counterfeit) [1]. It is reliant on the export nation’s ability to recover and allocate corneal tissue from within their donor pool.

Permitted corneal tissue transnational activity commenced in 1961 [3], out of the USA. To this day, the USA remains the most prominent routine exporter followed by Sri Lanka and Italy. Exportation is now credited for the provision for an estimated 23% all global transplants (being, n = 42,251 of n = 184 576 known annual global transplants in 2012) [4].

In contrast to larger export nations, Australia is not a routine exporter. Australia exports small quantities to New Zealand, and for occasional humanitarian requests within the Western Pacific and South East Asia regions [5].

With some activity already in existence in Australia, and with the potential to become routine exporters in the future [5,6], then examination of the practice is essential for current and future policy development. This is especially important given the potential sensitivities associated with the transfer of human biologicals internationally. Therefore, policy and regulatory reform relating to exportation must examine and incorporate the voice of the public, prior to implementing routine engagement. Therefore, the aim of our exploratory research is to understand the views of Australians and ascertain their willingness toward corneal export, by asking, should Australians export their cornea on their death, if the cornea was not needed in Australia at that time?.

While there has been some examination of transnational activity, e.g., recipient outcomes, when using exported corneal tissue^2^ and the opinion of the eye tissue and eye care sectors [7], unfortunately, there remains a paucity of information pertaining to donor understanding and knowledge of consent-for-export in Australia and elsewhere. There has been no research conducted to examine how populations feel about the concept of exporting their corneas and/or if they would consent for exportation on their death. Additionally, there is no global information to indicate how this activity functions or is monitored to ensure donor wishes are followed.

Our research, through examination of our own nation, Australia, captures for the first time a sample population’s opinion and intent to donate for exportation. It explores how and where Australians would like their donation to be used, and under what arrangements.

It is our intent, that our research would assist nations like Australia to consider if they should or should not export donated corneas. It provides foundation evidence required to build an export framework and implement a plan that matches the expectations of the Australian Public. Of note, our research does not examine if Australia is meeting domestic need or demand, how exportation could occur, or if Australia is ready to routinely do this. Instead, our focus is on unearthing the public’s opinion of a potential routine corneal tissue export option.

We acknowledge, that as Australia is not a routine exporter of corneal tissue, the Australian public and donors are not currently informed, consented or aware of the existence of the practice. There has been no discussion about the commencement of this and there are no details on Australian Eye Banks or affiliated government donation webpages or education materials, indicating that this practice of corneal tissue exportation occurs or may occur in the future. This means that for most respondents, our survey is probably the first time they have been asked to consider this concept.

Our null hypothesis was that Australians would be willing to donate corneal tissue for exportation. Additionally, we believed that the willingness of those born overseas–particularly in neighbouring countries, those with a vision impairment uncorrected by glasses, corneal recipients, those awaiting a corneal transplant, or those already registered on the nation’s Donatelife Register (https://donatelife.gov.au/register-donor-today) would support this notion more than other Australians. Lastly, our null hypothesised that those working in a health service would be more supportive of the concept than others due to familiarity with the health system, awareness of the benefits that can arise from transplantation and the known shortage in other countries. In terms of where they lived, and their age, gender, religion, or if they were Aboriginal or Torres Strait Islander, we had no hypothesis, and simply collected this information as a frequency descriptor only and thus this is exploratory in nature.

## Materials and method

Approval for this study was obtained from the Royal Victorian Eye and Ear Hospital’s Human Research Ethics Committee (HREC#18-1374H). Funding was provided by the Australian National Health and Medical Research Council and the Lions Eye Bank Western Australia to our lead researcher to conduct the study and manage the data.

Our e-survey was designed to examine a range of scenarios pertaining to the willingness of Australians to donate their corneal tissue to domestic and then international allocation. The scenarios presented in our final questionnaire (S1 Text), were devised through examination of transnationalism [2] and the collection of key themes and recommendations, from Australian and international eye care and corneal sector members [7]. We described corneal tissue transnational activity as donation sharing within the e-survey, to ensure that the respondents did not associate unethical trading or the black-market movement of human body parts and transplant tourism, with our examination of nationally planned and permitted activity. We also excluded words such as “trade” or “export”.

We designed and validated our survey tool (S1 Text), via a two-stage approach. During stage one, we devised and piloted our survey, ready for its use in a stage two formal e-survey with the Australian public. We describe our Stage 1 design method and validation in full, in S2 Text. Once our survey was validated, it was uploaded to the online survey tool, Qualtrics XM (USA)(https://www.qualtrics.com). Our formal e-survey took place in July 2019.

We selected Qualtrics because it provided stringent quality control features such as the ability to screen for dishonest, inaccurate, and speedy respondents, use of sophisticated digital fingerprinting to avoid duplication, and compliance with ISO standard and industry standard data protection and security procedures. Qualtrics already had a bank of potential respondents, and we wanted an e-interface that was already familiar to the respondent, so their time was spent considering the questions rather than managing the survey platform. As this was the first time a study of this nature, in this field, had been performed, and with no prior studies to follow or provide suggestions on the groups or types of respondents to engage, we sought a generalist response in order to start the process of long-term examination in this field of research. Therefore, we recruited respondents from an online panel as mentioned below.

### Participant recruitment

Qualtrics identified and recruited participants from their providers’ online panel, on the specifications of our selection inclusion criteria of Australian citizen or resident, aged 18 and over. Participants who completed the survey were provided with a small incentive. Our aim was to sample approximately 1000 individuals to achieve a margin of error of 3% at the 95% confidence level. Our intent was to select individuals to ensure that our sample group was representative of the current Australian population, based on the Australian Bureau of Statistics (ABS) 2016 Censuses [8] population demographics, and were relatively representative in terms of gender, age, state/territory population, Aboriginal and Torres Strait Islanders, and born outside of Australia rates. Through Qualtrics, n = 51,136 e-invitations were sent simultaneously to eligible participants. Those who were first to respond were able to participate. When the cohort was met, additional respondents were informed that they were not able to access the survey. The survey was closed at n = 1065 respondents.

We used the same consent process described in Stage 1, where participant consent was implied by their decision to click-ahead from the survey home page, to question 1 on the next page. The home page provided information on the survey, the research project and the stakeholders. It contained a pdf. downloadable information sheet explaining the research in full and included contact information. Finally, as the subject matter discussed death and donation which may be confronting for some, we provided contact details for *LifeLine*, a free counselling service in Australia.

### Analysis and sample size calculation

Responses were downloaded from Qualtrics, stored on a password protected server at the Centre for Eye Research Australia, then uploaded and analysed using IBM SPSS Statistics, version 26, software system (USA). Descriptive statistics determined the level and strength of the responses and confirmed our sample size was comparable to the ABS census data. Chi-Square and bivariate correlation coefficients (Pearson’s) examined associations between categorical variables. A sample size of 1000 provided ample statistical power for the analysis to detect an effect of the independent variables on the dependent variable, assuming the power of 0.80, with a small effect size and alpha of p = 0.05.

For the qualitative analysis, two open text boxes were included in the survey. The first asked those who had not decided to allow their corneas to be exported, what additional information they would require in order to make a decision (Q22a in S1 Text). The second provided an opportunity for participants to leave a final comment (Q25 in S1 Text). Responses were collated, consolidated and key themes identified based on overall frequency of responses.

## Results

### Respondents

A total of n = 1065 responses were received. We removed those that were incomplete or not completed appropriately (e.g., extensive data or response sets missing). This resulted in a final cohort of n = 1044 cleared responses.

According to Table 1, a total of 74.2% (n = 773) indicated they were born in Australia. The remaining 25.8% (n = 269) emigrated to Australia between 1949–2019 (with 1990 the mean). A total of n = 56 nations were represented, with the UK (n = 77), New Zealand (n = 29), India (n = 24), Germany (n = 13), Malaysia (n = 11), The Philippines (n = 11) and Indonesia (n = 9) dominant. Additionally, 44.4% (n = 464) of all respondents indicated that they had ties to relatives and friends outside of Australia.

**Table 1:** Participant demographics.

Willingness to export based on characteristics (%,n)		Significance *p* = .05	Willingness toward export		
			Yes %(n)	No %(n)	HND %(n)
**Gender**					
Female (49.2, 514)		0.54	38.5 (198)	22.1 (114)	39.2 (202)
Male (50.6, 529)			37.4 (198)	25.3 (134)	37.2 (197)
Other (0.09, 1)			100(1)	0(0)	0(0)
**Where they lived (State/Territory)**					
Australian Capital Territory (2.1, 22)		0.628	45.5 (10)	18.2 (4)	36.4 (8)
Northern Territory (0.9, 9)			33.3 (3)	33.3 (3)	33.3 (3)
New South Wales (30.2, 315)			34.3 (108)	25.1 (79)	40.6 (128)
Queensland (20.6, 215)			42.3 (91)	20.5 (44)	37.2 (80)
South Australia (7.2, 75)			44.0 (33)	29.3 (22)	26.7 (20)
Tasmania (2, 21)			47.6 (10)	14.3 (3)	38.1 (8)
Victoria (25.8, 269)			35.3 (95)	24.9 (67)	39.8 (107)
Western Australia (11.3, 118)			39.8 (47)	22 (26)	38.1 (45)
**Religion**					
Buddhist (1.8, 19)		0.539	42.1(8)	26.3(5)	31.6(6)
Christian (48.8, 509)			35.4(180)	26.5(135)	38.1(194)
Hindu (2.6, 27)			51.9(14)	14.8(4)	33.3(9)
Jewish (23, 9)			22.2(2)	33.3(3)	44.4(4)
Muslim (2.3, 23)			43.5(10)	30.4(7)	26.1(6)
No Religion (39.4, 411)			40.4(160)	20.2(83)	39.4(162)
Other (4.4, 46)			37.0(17)	23.9(11)	39.1(18)
**Age**					
15–25 (8.8, 92)		0.275	39.1(36)	15.2(14)	45.7(42)
26–35 (21.6, 226)			44.2(100)	22.1(50)	33.6(76)
36–45 (15.6, 163)			40.5(66)	23.9(39)	35.6(58)
46–55 (13.8, 144)			31.3(45)	25.0(36)	43.8(63)
56–65 (18.3, 191)			34.6(66)	24.1(46)	41.4(79)
66–75 (17.5, 183)			37.7(69)	27.9(51)	34.4(63)
76–85 (4.2, 44)			34.1(15)	27.3(12)	38.6(17)
86–95 (0.1, 1)			0.0(0)	0.0(0)%	100.0(0)
**Other Characteristics**					
Aboriginal and Torres Strait Islander (2.3, 24)		0.34	41.7(10)	33.3(8)	25(6)
Not Aboriginal and Torres Strait Islander (97.7, 1019)			37.9(386)	23.6(240)	38.6(393)
Born in Australia (74.2, 773)		0.072	37(286)	25.5(197)	37.5(290)
Not born in Australia (25.8, 269)			40.9(110)	18.6(150)	40.5(109)
Healthcare worker (92, 960)		0.104	37.1(356)	24.1(231)	38.9(373)
Not a healthcare worker (8, 84)			48.8(41)	20.2(17)	31.0(26)
Vision impairment not corrected by glasses (13.8, 144)		0.377	39.6(57)	27.1(39)	33.3(48)
No vision impairment or impairment corrected by glasses (86.2, 898)			37.6(338)	23.3(209)	39.1(351)
Received a corneal transplant (1, 10)		0.087	70(7)	20(2)	10(1)
Has not received a corneal transplant (99, 1034)			37.7(390)	23.8(246)	38.5(398)
Waiting for a corneal transplant (0.5, 5)		0.118	80(4)	20(1)	0(0)
Not waiting for a corneal transplant (99.5, 1039)			37.8(393)	23.8(247)	38.4(399)
Registered on the Donatelife Register (40.5, 422)		< .001	51.2(216)	18.5(78)	30.3(128)
Not registered on the Donatelife Register (59.5, 620)			29(180)	27.3(169)	43.7(271)
Intent to donate eyes (domestically) (35.6, 371)		< .001	58.8(218)	16.2(60)	25.1(93)
No intent to donate eyes (domestically) (18.3, 191)			16.2(31)	51.3(98)	32.5(62)
HND on intent donate eyes (domestically) (46.1, 481)			30.6(147)	18.7(90)	50.7(244)
	Intent to donate cornea for transplantation domestically (91.6, 340/371)	0.612	59.7(203)	15.3(52)	25(85)
	No intent to donate cornea for transplantation domestically (1.9, 7/371)		42.9(3)	28.6(2)	28.6(2)
	HND on donate cornea for transplantation domestically (6.5, 24/371)		50(12)	25(6)	25(6)

Demographics of the n = 1044 participants, including demographic and characteristic significance of their willingness to export their corneas (yes, no and had not decided = HND), analysed through Chi-Square and bivariate correlation coefficients (Pearson’s) that examined associations between categorical variables, with a sample size of n = ’1044, power of 0.80, and alpha of p = 0.05. (No minors were involved in this study).

When ocular history was considered, 13.8% (n = 144), indicated that their vision was not corrected by glasses. Their explanations ranged from old age, macular degeneration, retinal scaring, cataracts, short sightedness, astigmatism and some were unsure, however we identified that not all respondents answered correctly, with some saying they wore glasses for reading and driving–which indicated their vision was corrected with glasses. Only 1% (n = 10) indicated they had had a corneal transplant, with 0.5% (n = 5) indicating they awaited a corneal transplant. In comparison 4.4% (n-46) had known a relative who had had a corneal transplant, and 2.5% (n = 26) indicated they knew a relative who awaited a corneal transplant.

Table 1 also shows that 92% (n = 960) indicated that they worked in health care. Of those, n = 77/960 (8%) outlined their profession as: nurse/midwife (33.7%, n = 26), assistant/orderly (22%, n = 17), allied health (19.5%, n = 15), medicine/dental (10.2%, n = 8), administration/support (9.1%, n = 7) and medical scientist/pathology (5.25%, n = 4). The remaining 92% (n = 883/960) did not state where they worked in healthcare, making it difficult to ascribe a single work category.

### Willingness towards general donation and the Donatelife Register

**Fig 1:**
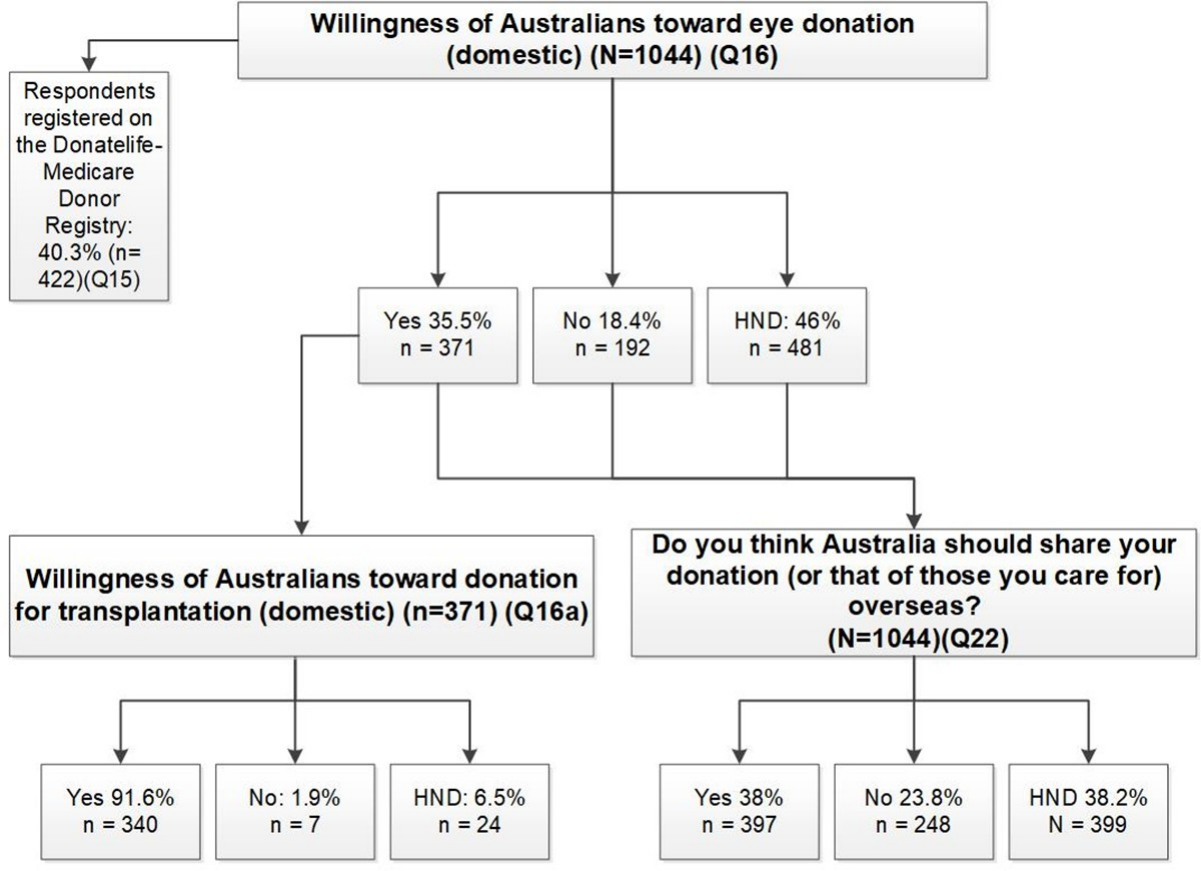
Willingness of Australian respondents towards general organ and tissue donation and then corneal donation domestically, and their willingness to export their corneal donation (yes, no that they had not decided = (HND) (Q = question).

Of our cohort, n = 40.5% (n = 422) indicated that they were already registered on the Donatelife Register (Q15 in S1 Text). This rate was higher than the 35% recorded registrants listed by the Australian Organ and Tissue Authority. Despite this, only 35.5% (n = 371) of respondents indicated a willingness to donate their eyes (Q16 in S1 Text). Fig 1 shows that of those, 91.6% (n = 340/371) indicated a willingness to donate for transplantation domestically (Q16a in S1 Text).

### Willingness to export CT

When specifically asked if Australia should export their corneas on their death, (Q22 in S1 Text), 38% (n = 397) indicated yes, 23.8% (n = 248) indicated no and the remaining 38.2% (n = 399) had not decided (See Fig 1).

Our analyses found no significant relationship between respondent demographics and characteristics of gender, age, where they lived, vision impairment, Aboriginal and Torres Strait Islander, transplant recipient, waiting for a corneal transplant, born in Australia, or healthcare worker, and their willingness to export (Table 1). Conversely, we did find a significant relationship between willingness to export and those who had registered on the Donatelife Register (p = < .001)(Q15 S1 Text), and those willing to donate their eyes domestically (p = < .001 (Q16 S1 Text). Significance did not extend to the sub-group of those who said yes to donation for transplantation domestically (Q16a in S1 Text) (p = .612). (See Table 1).

### Qualitative commentary

Those who had not decided on exportation were asked to explain their reservations. We identified 5 key themes, from their commentary, being: (1) don’t know, (2) need time to think about it, (3) more information needed, (4) up to others to decide, and (5) general concern (see Table 2).

**Table 2:** Respondent reservations on exporting their corneas.

Theme	Overview of comments
Don’t know	I didn’t know this was an option/Don’t know what questions to ask.
Need time to think about it	I need to discuss with my family/Depends how they feel at the time/I think I’m just avoiding the uncomfortable topic. On the one hand, I’m dead and my organs will decay anyway. On the other hand, something about it still makes me uncomfortable. I guess I assumed it would be something I’d deal with when I am old./Reluctant to think about being dead.
More information needed	How it will be used or decided/How can it be done without waste or damage/What is the success rate in other countries when using imported corneal tissue/A guarantee it gets there/Concern other country will sell it/More information.
Up to others to decide	Up to my family/The professionals will know what to do with it.
Concern	It’s scary/I would want to know the criteria, e.g. is it for profit?/Going to the rich not the poor.

Overview of the 5 key reservations of the 38.2% (n = 399) respondents that had not decided on exporting their corneas.

On the completion of the survey, respondents were offered an opportunity to provide a closing comment, with n = 163/1044 selecting to do so. The majority of comments were from those willing to export (n = 73/168, being 18.4% 73/397 of total ‘yes’ group). We summarise, their responses in Table 3, categorised based on their response to exportation.

**Table 3:** Respondent closing commentary.

Response (%, n/N = 1044)	Overview of comments
Yes (18.4, n = 73/397)	Your survey has made me think about this issue/Should go to the most needy—anywhere in the world/Simply put, I became an organ donor to enhance or save the life of fellow human beings, and it’s never mattered in any way where the recipient(s) come from.
No (13, n = 32/248)	Not donating my eyes/I will not be donating any of my body to anyone/Should look after our own country only/It’s quite eerie to think about it/I have strong values/Don’t want them sold or going overseas.
HND (15.8, n = 63/399)	I will leave it to my family and professionals to decide/I don’t know enough/As long as it’s not sold for money (apart from reimbursement for freight) it doesn’t bother me where it goes/So long as it was done ethically and morally I would have no issue.

Overview of the closing commentary from respondents who said yes or no to exportation or who had not decided (HND).

## Discussion

Our study indicated there are more Australians (38% n = 397) willing to export their corneas than not (23.8%, n = 248). The ‘yes’ group had few reservations about the process, viewing it as a humanitarian act. They recognised it had the potential to assist recipients of other countries who awaited a corneal transplantation and would otherwise be unable to receive such treatment without their donation. While this supports our exploration of a routine export system for Australia, further planning and information would be required prior to implementation. For example, a consent-for-export mechanism would assist in ensuring that those wishing to export had the option to do so.

Conversely, some respondents (23.8%, n = 248) said “no” to exportation. They were predominantly concerned that Australian recipients in need may be neglected or the corneal tissue may be misused or wasted when exported. Their concerns are currently valid as there is no public information explaining how the process would occur, and they therefore have minimal information to guide their decision making. This indicates, that if Australia were to proceed with developing a routine export system, then it should provide information that describes the process. A withdrawal mechanism within a consent-for-export process would also ensure the wishes of those that declined, were respected and met, and their donations were retained in Australia. We wonder, however, if individuals in this group would be willing to export once information regarding the practice was readily available and clearly explained.

Interestingly, 38.2% (n = 399) indicated they had not decided. This was the largest responder group. This represented an important group to emphasise because, as previously described, at the time of our e-survey, corneal tissue exportation was not common practice in Australia and information regarding the existence was not provided in pre-consent information; nor in public campaigns or on websites, nor at the point-of-donation. This meant the Australian population, in general, has not been presented with the facts necessary to make a decision. This group represented a core group of Australians who, once informed appropriately, could respond either yes or no. Moreover, Australians in this group may be willing to export if fully informed. The qualitative results from these respondents indicated that more information prior to the point-of-donation was necessary for their decision making.

Our results also indicated that before Australia routinely exports corneas, further examination of Australian’s willingness to export may be required. For example, would the next-of-kin in real-world situations permit the exportation of their loved ones’ cornea? Additionally, a carefully coordinated and crafted national and/or jurisdictional public education campaign may be required to present information about exportation, clearly indicating how the process works. It could be monitored, and the options made available to donors. This would assist members of the public in their decision making, well before the time of donation, when donor families are required to make a quick decision. Such a campaign may also require examination of the consent process, with consent-for-export a potential inclusion. Transparency, and the inclusion of consent-for-export are particularly important for the ‘no’ group who may adamantly insist that their donation is not exported. Without communicating or providing an option to opt-in or out, this may unintentionally defy the wishes of the donor, undermine the public’s trust in the sector, and in turn their willingness to donate for a range of needs in the future. This may directly impact recipients in Australia, and beyond, who require a corneal transplant.

While our research demonstrates that a third of Australians are willing to donate, we did not find significant differences in willingness amongst respondents based on demographic and characteristic lines. Our only exception was in relation to those already registered on the Donatelife Register and those who had already decided their own end-of-life donation for transplantation decision. Perhaps these groups, more than others, had already been exposed to the general concept of donation, and were more familiar and aware of how the system and sector functioned in Australia. Making the leap from just domestic to international, may have been less confronting to these respondents possibly due to their existing knowledge. As the current Australian system does not provide details on where Australian donations are currently allocated to (e.g., it does not indicate that donations may be allocated in other parts of Australia either), then perhaps this group did not mind where the donation would be sent, and instead focused more on the act of donation as a wholistic act based on the premise of helping others. As this group had pre-determined their donation decision, this also supported the premise that informing the public about donation, allowing them to consider their donation in full before their death and the moment of donation, is a powerful motivator in their decision making.

### Limitations

While using an online panel sample method allowed a baseline understanding of the populations’ opinion, we acknowledge that those who participated were incentivised to participate, though as our questions presented new concepts, we do not believe it influenced our outcomes one way or another. Using another sampling method in the future may offer a comparison. Additionally, the methodology used did not allow for in-depth analysis of why respondents selected to export or not. Further research e.g., focus, or democracy groups may provide greater detail in the future, and could unearth opinion, influences on opinion, and transition group respondents from one viewpoint to another, once presented with the facts about exportation.

While we found no significance in willingness from health professionals in our sample group, as 92% of our participants indicated they were healthcare workers, the results may propose a bias in their response. Therefore, future studies comparing health care professional and non-health care professional group motivation may explain our findings. Future research could also examine sub-categories such as socio-economic groups and education level.

With no prior examination of this subject matter, we have no way of knowing if the Australian population responded similarly to other populations, how they compared with nations that do routinely export or if this is consistent with their opinion about the exportation of other human biologicals (e.g., blood, bone, reproductive materials). As the first survey of this nature, we cannot determine how changes in opinion or willingness have altered over time, however our research can be used as a baseline indicator to help gauge adaption going forward. Further comparative studies of this nature could contextualise our Australian responses, and may offer nations, particularly those already exporting, the opportunity to examine, revise and improve their services and transparency with their own population. Our sample size was large enough to suggest that several sub-characteristics were not significant to our respondents’ willingness to export (e.g. gender, vision impairment, born overseas), though we wonder if, via focus or democracy groups, this could be examined in more detail.

### Recommendation

If Australia decided to routinely export, then informing the public before the point-of-donation, indicating that exportation is an option, is an essential planning feature. We also recommend that donors are consented specifically for exportation in a similar manner to consenting for research. It is important that public messages in this regard, are carefully crafted so exportation messaging does not undermine existing domestic messaging. The eye tissue, donation and eye care sectors would also need to develop an allocation system that worked in unison to ensure Australian domestic demand (being transplant, training and research) was catered for simultaneously to export demand. Finally, Australia would need to clearly explain the process, e.g., how, and why they donate to particular countries, and under what arrangements. Such mechanisms could include:

1. Tick-box added to the Donatelife Register asking donors to indicate their willingness to donate for export if domestic demand was met at the time of their death.
  - This could be accompanied with an explanation of why this system exists and how export locations are selected and monitored.
2. Provision of public information, e.g.:
  - Eye banks, peer associations etc. indicating on their website that they participate in exportation and may export if domestic demand was met at that time. This is accompanied by an indication of how they determine their export partners.
3. Routinely offer Australian donors the option to consent-for-export, by:
  - Updating the donor consent process and forms to include export information to capture consent.
  - Donor coordinators/eye bankers incorporate consent-for-export into their point-of-donation conversation.
4. Implement a nationally coordinated system to ensure exported corneal tissue are allocated, tracked, monitored and reported nationally, by:
  - Ensuing transparency and safeguards are in place to protect the Australian donation.
  - Development of export allocation criteria.

We reiterate that the benefits of the Donatelife Register and the pre-death chance to consider the options were key motivators in our respondent’s willingness to export. We cannot underestimate the value of informing populations prior to the point-of-donation. It alleviates concerns, allows for questions to be answered, and ensures the system is accountable and trackable. It removes taboos such as trafficking and profit which are independent factors not necessarily related to exportation but often assumed or confused as one in the same. By presenting the information, and removing the taboos, Australians would have access to the necessary information they need to make their own decision.

To close, our research suggests that a significant proportion of Australians are willing to export their end-of-life corneal donation. Conversely, there are Australians not willing to participate, or are undecided. Collectively, they warrant discussion on the development of a formal and transparent export system, inclusive of a consent-for-export opt-in or opt-out option. Lastly, developing such a system for Australian corneal tissue, may assist other nations and tissue type custodians who may also wish to examine their export process and population willingness, and ultimately ensure donor wishes are respected.

## Supporting information

S1 Text E-survey: Sharing donated corneas overseas. (DOCX)

S2 Text Methodology and validation process. (DOCX)
