# Supplementary material for: Determining the willingness of Australians to export their corneas on death
Source: PLoS One. 2021 Feb 19;16(2):e0246622. doi: 10.1371/journal.pone.0246622 (PMC7894941; doi:10.1371/journal.pone.0246622)
Supplement: S1 Text — (DOCX) [file pone.0246622.s001.docx]

Supplement 1: E-survey: Sharing donated corneas overseas

**This document**

This document outlines the screen/display pathway of the e-survey. All participants must complete the consent process prior to being directed to the e-survey. An automated thank you will appear after the submission of the last question.

*Not seen by the participant:*

- Grey boxes: The boxes indicate where the question has been allocated an action, e.g. end of question, skip questions, additional questions.
- Sections and page breaks: These indicate where a new section appears to the participant.
- Numbers in brackets after text options: This is for tracking purposes.

**Consent**
 [Information provided before commencement].

**Consent to Participate**

I have read/or have had read to me in a language that I understand, the above/attached information, and I understand the purposes, procedures and risks of this research project as described within it. I confirm that I am an Australian citizen or permanent resident over the age of 18 years. I have had an opportunity to ask questions and I am satisfied with the answers I have received. I freely agree to participate in this project according to the conditions outlined above. The researcher has agreed not to reveal my identity and personal details if information about this project is published or presented in any public form without permission

- Yes (1)
- No (2)

Skip To: End of Survey If Information to read before consenting Consent to Participate I have read/or have had read to me in... = No

| Page Break |  |
| --- | --- |

Q1 Are you a permanent resident or citizen of Australia?

- Yes (1)
- No (2)

Skip To: End of Survey If Are you a permanent resident or citizen of Australia?  = No

Q2 Where do you live?

- Queensland (1)
- South Australia (2)
- Tasmania (3)
- Victoria (4)
- Northern Territory (5)
- New South Wales (6)
- Western Australia (7)
- Australian Capital Territory (8)

| Page Break |  |
| --- | --- |

Q3 Gender

- Male (1)
- Female (2)
- Other (3) ________________________________________________

| Page Break |  |
| --- | --- |

Q4 Age

- Type in age (1) ________________________________________________

| Page Break |  |
| --- | --- |

Q5 Religion

- Christian (1)
- Jewish (2)
- Muslim (3)
- Hindu (4)
- Buddhist (5)
- No religion (6)
- Other (7)

| Page Break |  |
| --- | --- |

Q6 Do you work in the healthcare or medical science sector?

- Yes (2)
- No (1)

Display This Question:

If Do you work in the healthcare or medical science sector? = Yes

Q6a Please outline if this is as a doctor, nurse etc. and which sub-specialty

________________________________________________________________

| Page Break |  |
| --- | --- |

End of Block: Section 1: In this section we will be asking general questions about yourself.

Start of Block: Eye and transplantation

Q7 Are you of Aboriginal or Torres Strait Islander descent?

- Yes (1)
- No (2)

Display This Question:

If Are you of Aboriginal or Torres Strait Islander descent?  = No

Q7a How do you ethnically identify? (e.g. White European, Indo-Chinese)

________________________________________________________________

Q8 Were you born in Australia?

- Yes (1)
- No (2)

Display This Question:

If Were you born in Australia?  = No

Q8a Which country were you born in?

________________________________________________________________

Display This Question:

If Were you born in Australia?  = No

Q8b Which year did you move to Australia?

________________________________________________________________

Q9 Do you have relatives/close friends living in another country?

- Yes (2)
- No (1)

Display This Question:

If Do you have relatives/close friends living in another country?  = Yes

Q9a Please indicate which countries

________________________________________________________________

| Page Break |  |
| --- | --- |

Q10 Do you have a vision impairment that is not corrected by contact lenses or glasses?

- Yes (2)
- No (1)

Display This Question:

If Do you have a vision impairment that is not corrected by contact lenses or glasses?  = Yes

Q10a Please describe your vision impairment

________________________________________________________________

| Page Break |  |
| --- | --- |

Q11 Have you been a recipient of a transplant involving human donated eye tissue?

- Yes (1)
- No (2)

Q12 Are you awaiting a transplant involving human donated eye tissue?

- Yes (1)
- No (2)

Q13 Has a close relative/someone you’ve cared for been a recipient of a transplant involving human donated eye tissue?

- Yes (1)
- No (2)

Q14 Is a close relative/someone you’re caring for awaiting a transplant involving human donated eye tissue?

- Yes (1)
- No (2)

| Page Break |  |
| --- | --- |

Q15 Have you registered yourself on the Australian Medicare, DonateLife Registry to be a donor on your death?

- Yes (1)
- No (2)

Q16 Regardless of being on the Donor Registry, are you intending to donate your eyes on your death?

- Yes (1)
- No (2)
- Haven't thought about it (3)

Display This Question:

If Regardless of being on the Donor Registry, are you intending to donate your eyes on your death?  = Yes

Q16a Do you intend to consent to donate for transplantation?

- Yes (1)
- No (2)
- Haven't thought about it (3)

Display This Question:

If Regardless of being on the Donor Registry, are you intending to donate your eyes on your death?  = Yes

Q16b Do you intend to consent to donate for research and/or training?

- Yes (1)
- No (2)
- Haven't thought about it (3)

| Page Break |  |
| --- | --- |

Q17 Have you ever been the end-of-life next-of-kin (primary carer) to someone else?

- No (1)
- Yes (3)
- Prefer not to answer (4)

Display This Question:

If Have you ever been the end-of-life next-of-kin (primary carer) to someone else?  = Yes

Q17a Were you approached to discuss donation of their eyes, on their behalf?

- Yes (2)
- No (1)
- Can't remember (3)
- Prefer not to say (4)

Display This Question:

If Were you approached to discuss donation of their eyes, on their behalf?  = Yes

Q17b Did you consent to allow the donation of their eyes?

- Yes (1)
- No (2)
- Can't remember (3)

Display This Question:

If Did you consent to allow the donation of their eyes? = Yes

Q17c Please share:

|  | Yes (1) | No (2) | Can't remember (3) |
| --- | --- | --- | --- |
| Did the donation take place? (1) |  |  |  |
| Was the consent for transplantation? (2) |  |  |  |
| Was the consent and/or for research and training? (3) |  |  |  |

| Page Break |  |
| --- | --- |

End of Block: Eye and transplantation

Start of Block: Section 2: In this next Section, we will be asking your opinion regarding if and

Q18 Please indicate where you would be happy for your donation to go, to assist those in need of an eye transplant. You may select more than one option.

|  | Yes (1) | No (2) | I will leave it to the professionals to decide where it’s needed (3) | Haven't thought about it (4) |
| --- | --- | --- | --- | --- |
| Your local town/city (1) |  |  |  |  |
| Your State/Territory (2) |  |  |  |  |
| Australia (3) |  |  |  |  |
| New Zealand (4) |  |  |  |  |
| Neighbouring countries of Asia-Pacific (5) |  |  |  |  |
| Commonwealth Countries (6) |  |  |  |  |
| Asia Pacific Economic Countries (APEC) Countries (7) |  |  |  |  |
| Any country where there is evidence of need (8) |  |  |  |  |
| Specific Country/Other (9) |  |  |  |  |

Q19 Please indicate where you would be happy for the donation of those in your care to go, to assist those in need of an eye transplant. You may select more than one option.

|  | Yes (1) | No (2) | I will leave it to the professionals to decide where it’s needed (3) | Haven't thought about it (4) |
| --- | --- | --- | --- | --- |
| Your local town/city (1) |  |  |  |  |
| Your State/Territory (2) |  |  |  |  |
| Australia (3) |  |  |  |  |
| New Zealand (4) |  |  |  |  |
| Neighbouring countries of Asia-Pacific (5) |  |  |  |  |
| Commonwealth Countries (6) |  |  |  |  |
| Asia Pacific Economic Countries (APEC) Countries (7) |  |  |  |  |
| Any country where there is evidence of need (8) |  |  |  |  |
| Specific Country/Other (9) |  |  |  |  |

| Page Break |  |
| --- | --- |

Q20 In the event that Australia was meeting surgical eye transplant needs, at the time of your death, how would you prefer your donation to be used? You may select more than one.

|  | Yes (1) | No (2) | I will leave it to the professionals to decide where it’s needed (3) | Haven't thought about it (4) |
| --- | --- | --- | --- | --- |
| Stay in Australia to train Australian surgeons (1) |  |  |  |  |
| Stay in Australia to assist Australian Research (2) |  |  |  |  |
| Go overseas to help a person in need of a transplant (3) |  |  |  |  |
| Go overseas to help another country to train their surgeons (4) |  |  |  |  |
| Go overseas to help research in another country (5) |  |  |  |  |
| Withdraw the donation (6) |  |  |  |  |

| Page Break |  |
| --- | --- |

Q21.       In the event that Australia was meeting surgical eye transplant needs, at the time of death of those in your care, how would you prefer your donation to be used? You may select more than one.

|  | Yes (1) | No (2) | I will leave it to the professionals to decide where it’s needed (3) | Haven't thought about it (4) |
| --- | --- | --- | --- | --- |
| Stay in Australia to train Australian surgeons (1) |  |  |  |  |
| Stay in Australia to assist Australian Research (2) |  |  |  |  |
| Go overseas to help a person in need of a transplant (3) |  |  |  |  |
| Go overseas to help another country to train their surgeons (4) |  |  |  |  |
| Go overseas to help research in another country (5) |  |  |  |  |
| Withdraw the donation (6) |  |  |  |  |

| Page Break |  |
| --- | --- |

Q22 Do you think Australia should share your donation (or that of those you care for) overseas?

- Yes (1)
- No (2)
- Haven’t made up my mind (3)

Display This Question:

If Do you think Australia should share your donation (or that of those you care for) overseas?  = Haven’t made up my mind

Q22a please outline your reservations and/or what further information you require in order to support your decision either way.

________________________________________________________________

| Page Break |  |
| --- | --- |

End of Block: Section 2: In this next Section, we will be asking your opinion regarding if and

Start of Block: Section 3: You have now completed the e-survey. In this section, we ask you to r

Q23 If Australia was able to provide enough corneas to meet the surgical need within Australia, and did not need your eye donation (or that of those you care for) at the time, and was to share your donation with other nations, please indicate what process and allocation steps you would like the Australian donation and eye care professionals to consider and/or prepare, prior to doing so. The Donation and Recovery Process:

|  | Yes (1) | No (2) | I will leave it to the professionals to decide where it’s needed (3) | Haven't thought about it (4) |
| --- | --- | --- | --- | --- |
| Provide information regarding overseas sharing on the DonateLife/Medicare/Eye Bank websites (1) |  |  |  |  |
| Place a tick box on the Donor Registry, indicating your intent to share overseas in the event that Australia did not need it at that time (2) |  |  |  |  |
| Include overseas sharing information in the face-to-face conversation, with hospital staff/eye bank staff, during the end-of-life consent and decision-making process (3) |  |  |  |  |
| Place a tick box on the consent form, indicating willingness to share overseas or retain in Australia (4) |  |  |  |  |

Q24 If Australia was able to provide enough corneas to meet the surgical need within Australia, and did not need your eye donation (or that of those you care for) at the time, and was to share your donation

with other nations, please indicate what process and allocation steps you would like the Australian donation and eye care professionals to consider and/or prepare, prior to doing so.

This section examines the eye care allocation process (Please indicate how you would like Australia to decide on who to share your donation with):

|  | Yes (1) | No (2) | I will leave it to the professionals to decide where it’s needed (3) | Haven't thought about it (4) |
| --- | --- | --- | --- | --- |
| Countries Australia has a government humanitarian aid relationship with (AusAid) (1) |  |  |  |  |
| Countries where humanitarian eye care providers, recognised by Australia, have evidence of existing eye care training and infrastructure programs (2) |  |  |  |  |
| Countries where Australian eye transplant surgeons provide voluntary surgery or training (3) |  |  |  |  |
| Countries able to reimburse Australia the processing and freight costs (mid-high-income countries) (4) |  |  |  |  |
| Countries unable to reimburse Australia, and require humanitarian assistance (low-mid income countries) (5) |  |  |  |  |
| Locations based on the practicalities of freight, logistics, time and tissue handling techniques (6) |  |  |  |  |
| Active conflict zones (7) |  |  |  |  |

Q25 Do you have any general comments or other suggestions regarding eye donation use in Australia or sharing it with waiting recipients in other nations in the event that Australia does not need it at that time?

________________________________________________________________

| Page Break |  |
| --- | --- |
