# Supplementary material for: Determining the willingness of Australians to export their corneas on death
Source: PLoS One. 2021 Feb 19;16(2):e0246622. doi: 10.1371/journal.pone.0246622 (PMC7894941; doi:10.1371/journal.pone.0246622)
Supplement: S2 Text — (DOCX) [file pone.0246622.s002.docx]

**Supplement 2:** **Methodology and validation process**

We commenced our research question design by completing a review of transnational activity.^1^ We then interviewed global eye care and eye tissue sector members to identify key themes.^2^ This was followed by the development and validation of our e-survey tool (Stage 1 – outlined in this Supplemental document) and finally our formal Qualtrics XM (USA) e-survey (Stage 2) as described in the main text (Diagram 1).


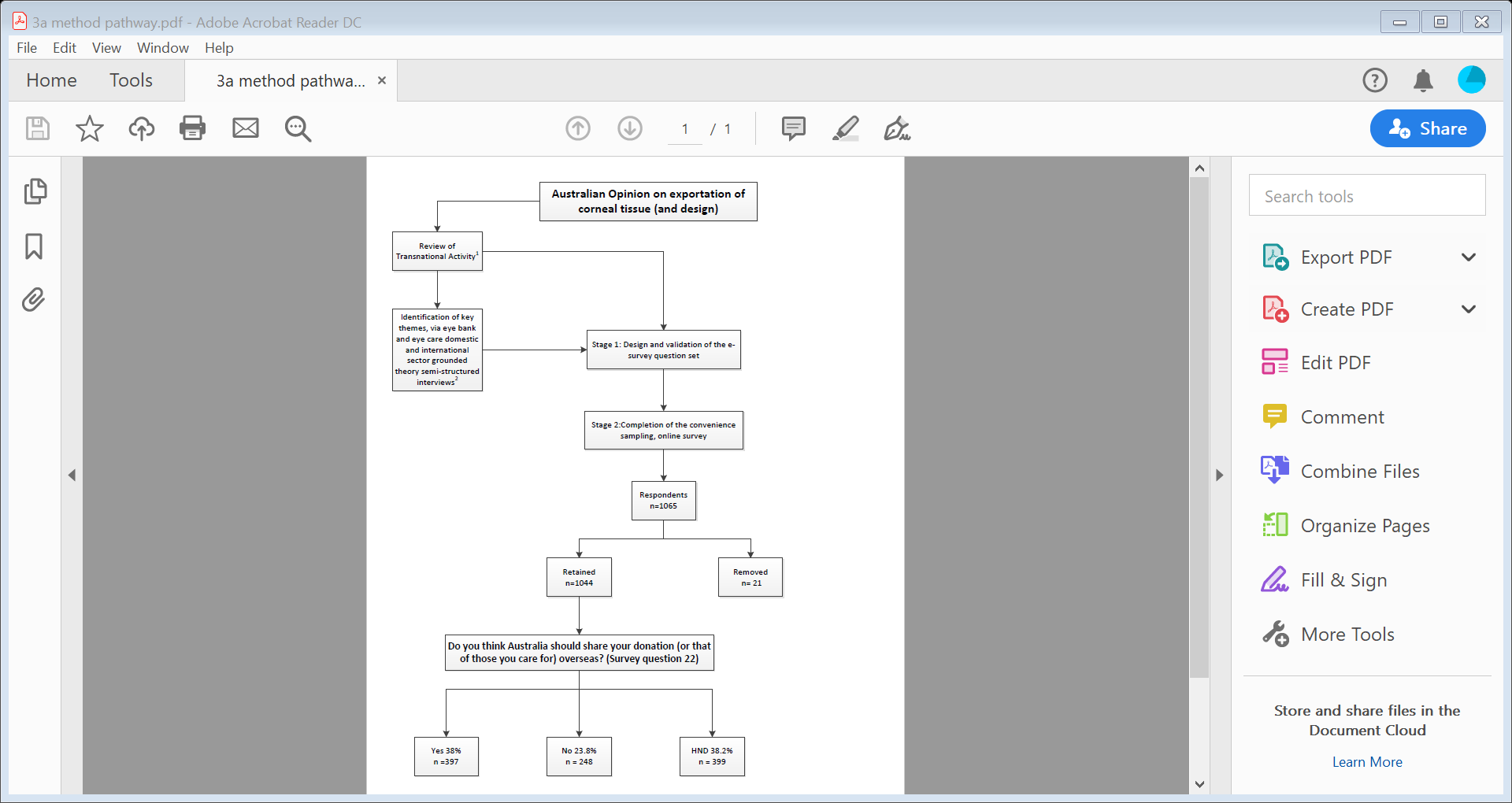


*Diagram 1:* The design, validation and research steps used to ascertain the willingness of Australians to export their corneal tissue donation on their death. *HND = Have not made up their mind.*

***Stage 1:*** Our e-survey was designed to examine a range of scenarios pertaining to the willingness of Australians to donate their corneal tissue for domestic and then international allocation. The scenarios (e.g. Question 22: Do you think Australia should share your donation (or that of those you care for) overseas?) presented in our final questionnaire (Supplementary 1), were devised through our examination of transnationalism^1^ and our collection of key themes and recommendations, from Australian and international eye care and corneal sector members.^2^

We devised our grounded theory pilot e-survey tool, and uploaded it to the Qualtrics XM survey online software tool. We described corneal tissue transnational activity as *donation sharing* within the e-survey, to ensure that the respondents did not associate unethical trading or the black-market movement of human body parts and transplant tourism, with our examination of nationally planned and permitted activity. We excluded words such as “trade” or “export”.

in total, n=13 purposively selected individuals not familiar with our project or field of work completed the pilot e-survey. Participant consent was presumed by their decision to click-ahead from the survey home page, to question 1 on the next page. The home page provided information on the survey, the research project and the stakeholders. It contained a pdf. downloadable information sheet that explained the research in full. The information sheet provided contact information for Qualtrics and our human research ethics committee. Finally, as the subject matter discussed death and donation, which may be confronting for some, we provided contact details for *LifeLine*, a free counselling service in Australia.

To ensure our questions were understood and answered correctly, our pilot group were additionally asked to provide feedback on the survey itself (e.g. the approach, respect for the subject matter, length, phraseology and so on). Their responses were supportive with no context changes identified, other than spelling or arrangement of the question order. Their feedback was incorporated and assisted in validating and finalising the e-survey. Stage 1 took place in June 2019.

*The e-survey:* The e-survey (Supplementary 1) was provided in English, with an estimated completion time of 6-10 minutes. Questions were designed to ensure relevant bio-psychosocial aspects (e.g. a recent death in the family) were captured and approached respectfully. The final e-survey involved 25 questions, 12 of which included sub-questions, that appeared depending on the respondent’s initial response. The e-survey commenced with standard demographic questions (e.g. age, gender). We added generalist questions relating to donation, vision impairment and donation allocation domestically and internationally. For example, we asked the participant if they had a vision impairment that could not be corrected by glasses, or if they had received a corneal transplant and so on. We included these as we wanted to understand if respondents who had found themselves in these situations had a greater or lesser affiliation to the sharing of donations outside of Australia. We hoped to determine if Australians with vision impairment, or those who had (or were awaiting) a transplant and those who knew someone who had donated, would have a greater or lesser emotive willingness to share and prevent suffering in others, regardless of their location. We also asked respondents if they were on the Donor Registry, if they were Aboriginal or Torres Strait Islander, born outside of Australia or had an affiliation with other nations through friends or family (as we wanted to examine if their connection to Australia and other nations increased or decreased their willingness to share their donation overseas). Finally, we asked if the respondent worked in the health or medical science field to determine if those within a related field had a greater or lesser response to the concepts we proposed.

We were aware that several of our questions would be new to the participants, as transnational activity is not widely discussed or understood in the Australian context and as such we did not expect our participants to have a definitive decision on their desire to export their donation or not, without having the opportunity to truly understand what was involved. We felt that by presenting them the questions, in this manner, it replicated the current Australian setting were donors are not aware, and as such would need to decide on the spot at the point of donation, if Australia were to commence exportation today, without any information provided. We believed this approach would highlight that donors do not have the full facts at their disposal at the point of donation to make a sound and informed decision. In our e-survey we ask donors specific questions, for example, can we export your corneal tissue if it is not needed in Australia? In this instance participants had the option to say Yes, No or had not decided.

**References:**

1. Machin H, Arslan J, Baird PN. Examining the impact of corneal tissue transnational activity, and transplantation, on import and export nations: a review of the literature. *Cornea*. 2020. 39(6):795-800.
2. Machin H, Sutton G, Baird PN. Should nations with surplus donated corneal tissue, export to those without? A review of sector opinion via the example of one nation – Australia. *Cornea.* In-print.
